# Microwave Metasurface-Based Sensor with Artificial Intelligence for Early Breast Tumor Detection

**DOI:** 10.3390/mi17020179

**Published:** 2026-01-28

**Authors:** Maged A. Aldhaeebi, Thamer S. Almoneef

**Affiliations:** Electrical Engineering Department, College of Engineering, Prince Sattam Bin Abdulaziz University, Al-Kharj 11942, Saudi Arabia; t.almoneef@psau.edu.sa

**Keywords:** meta-materials, metasurface, microwave sensors, artificial intelligence, detection, breast tumors

## Abstract

In this paper, a microwave metasurface sensor integrated with artificial intelligence (AI) for breast tumor detection is presented. The sensor’s sensitivity is estimated by analyzing shifts in magnitude and the phase of the reflection coefficient ($S_{11}$) obtained from normal and abnormal breast phantoms. The ($S_{11}$) responses of 137 anatomically realistic 3D numerical breast phantoms in standard classes, C1—mostly fatty, C2—scattered fibroglandular, C3—heterogeneously dense, and C4—extremely dense, incorporating different tumor sizes are used as input features. A custom neural network is developed to detect tumor presence using the recorded ($S_{11}$) responses. The model is trained with cross-entropy loss and the AdamW optimizer. The dataset is split into training (70%), validation (15%), and test (15%) sets. The model achieves 99% accuracy, with perfect precision, recall, and F1-score across individual classes. For paired class combinations, accuracies of 71% (C1 with C2) and 65% (C2 with C3) are obtained, while performance degrades to approximately 50% when all four classes are combined. The sensor is fabricated and experimentally validated using two physical breast phantoms, demonstrating reliable detection of a 10 mm tumor. These results highlight the effectiveness of combining microwave metasurface sensing and AI for breast tumor detection.

## 1. Introduction

Breast cancer remains one of the most significant causes of cancer-related deaths among women globally, underscoring the critical need for effective early detection strategies to enhance survival rates and reduce treatment costs [1]. Traditional screening methods such as mammography, ultrasound, and magnetic resonance imaging (MRI) are widely used; however, they are limited by issues related to sensitivity, specificity, and patient comfort [2]. For instance, mammography, while effective in many cases, struggles to detect tumors in dense breast tissue, often leading to false positives or false negatives [3] Additionally, MRI, although highly sensitive, is expensive, time-consuming, and not always accessible in low-resource settings [4]. These limitations highlight the growing demand for innovative diagnostic approaches that provide higher accuracy, non-invasive procedures, and cost-effectiveness.

Recent advancements in imaging technologies, such as digital breast tomosynthesis (DBT) and contrast-enhanced mammography (CEM), have shown promise in addressing some of these challenges by improving tumor detection rates and reducing diagnostic errors [5,6]. However, these methods still rely on ionizing radiation or contrast agents, which may not be suitable for all patients. In this context, microwave imaging (MI) has emerged as a promising alternative due to its non-ionizing nature, cost-effectiveness, and ability to differentiate between healthy and malignant tissues based on their dielectric properties [7,8,9]. Unlike traditional methods, MI is particularly effective in imaging dense breast tissue, where conventional techniques often fall short [10].

Microwave sensing has become an increasingly promising method for medical imaging, particularly as a complement to traditional breast cancer screening techniques. This technology works by utilizing electromagnetic waves that interact with biological tissues, which have distinct dielectric properties. These properties enable the detection of anomalies, such as tumors, because malignant tissues typically have different dielectric characteristics compared to healthy tissues. Furthermore, microwave imaging is less sensitive to variations in tissue density, making it an especially useful tool for women with dense breast tissue. Tumor tissues in the breast have significantly higher dielectric constants and electrical conductivities in the microwave frequency range when compared to normal tissues [7,11,12,13].

Recent research has seen a shift from traditional microwave imaging to microwave detection methods in the field of breast cancer diagnosis. While microwave imaging focuses on creating detailed images of the breast tissue, microwave detection aims to identify the presence of tumors by analyzing the unique dielectric properties of malignant tissues. This shift is driven by the need for more accurate, non-invasive, and cost-effective screening methods, particularly for women with dense breast tissue, where conventional imaging techniques like mammography may face limitations. Microwave detection has shown promise due to its ability to detect changes in dielectric constant and electrical conductivity in tumors, which differ significantly from surrounding normal tissue. This approach provides an efficient means for early tumor detection, offering higher sensitivity and specificity, especially in dense breasts [9,11,12,14,15,16,17,18,19].

The integration of artificial intelligence (AI) with microwave imaging has further enhanced its diagnostic potential. AI algorithms, particularly machine learning (ML) and deep learning (DL), can analyze complex microwave signals to improve image reconstruction, reduce noise, and enhance tumor localization [20,21]. These AI-driven approaches enable real-time analysis and decision making, making MI a viable tool for early and accurate breast cancer detection. Furthermore, numerical analysis of microwave sensors, combined with AI, allows for the optimization of sensor design and imaging protocols, ensuring higher precision and reliability in clinical applications [22].

This paper presents a novel method for breast tumor detection by combining microwave sensors with artificial intelligence (AI) algorithms. The proposed metasurface sensor, composed of 64 unit cells and powered by a single port, is designed to enhance detection capabilities. This sensor system is integrated with an AI model aimed at improving diagnostic accuracy by effectively analyzing the data collected. The primary goal of this work is to advance breast cancer diagnostics by offering a non-invasive, efficient, and accessible solution for early tumor detection, ultimately leading to better clinical outcomes and enhanced patient care.

## 2. Metasurface Sensor Design and Modeling

**Figure 1:**
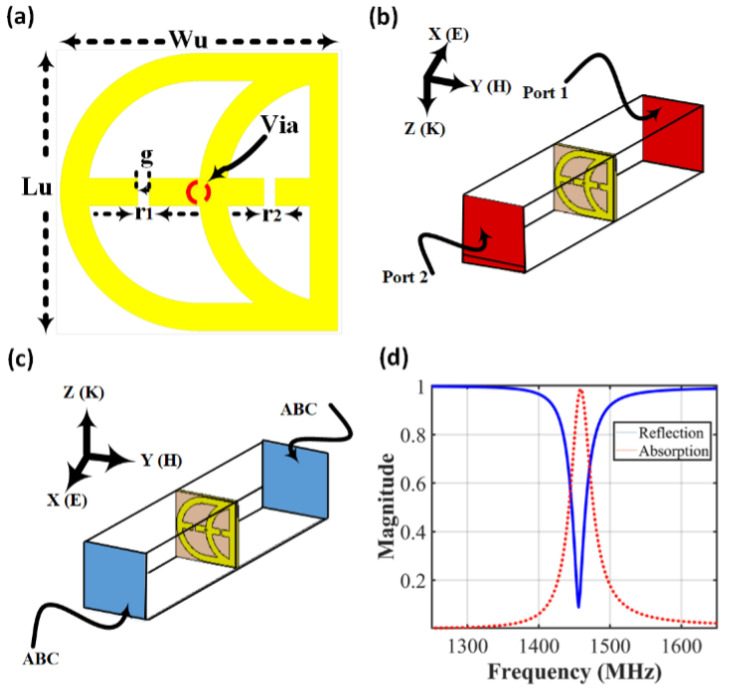
Proposed unit cell: (**a**) schematic of a single element of the proposed half-loop metasurface; (**b**,**c**) simulation setup of the metasurface sensor unit cell placed inside a waveguide, with periodic boundary conditions in the transmitting and receiving mode configurations, respectively; (**d**) simulated reflection and absorption coefficients of the unit cell.

The unit cell of the proposed metasurface sensor features a compact and optimized design consisting of two cascaded half-loops with an embedded dipole that includes two central gaps. This structure is built on a Rogers RO3010 dielectric substrate, which has a thickness of h=1.27mm, a dielectric constant of $\varepsilon_{r}=10.2$, and a loss tangent of tanδ=0.0022, supported by a reflector to improve the overall performance, as shown in Figure 1. The unit cell, designed to operate at f=1.48GHz, has the following dimensions: L=20.5mm, $d_{1}=3.5\;\mathrm{mm}$, $d_{2}=2.5\;\mathrm{mm}$, g=0.8mm, s=0.5mm, and a copper thickness of t=35 µm. A shunt-fed port with an impedance of $Z_{0}=160\;\Omega$ connects the top and bottom layers via to via, enabling efficient signal transmission. The design was simulated and optimized using CST Microwave Studio, ensuring reliable performance for its intended application [23]. The unit cell was intentionally designed to provide dual sensitivity to the primary electromagnetic contrasts between healthy and malignant breast tissues. The structure integrates an embedded dipole with central gaps and cascaded half-loop elements. The central gaps in the dipole act to concentrate the electric field, thereby enhancing sensitivity to variations in the effective dielectric constant of the surrounding tissue. In parallel, the cascaded half-loops increase the electromagnetic interaction volume with the adjacent medium, which improves sensitivity to conductivity-related losses. The combination of these complementary mechanisms enables the metasurface sensor to respond effectively to tumor-induced changes in both dielectric permittivity and conductivity, supporting reliable detection of breast tissue abnormalities.

**Figure 2:**
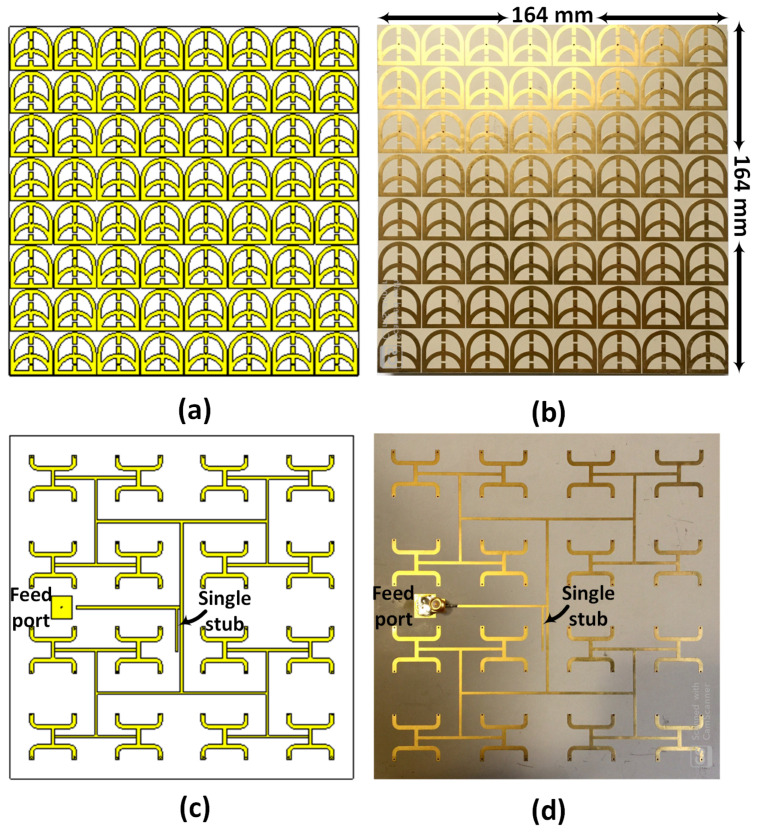
Photographs of the proposed half-loop metasurface array sensor: (**a**) top view of the design layout showing the array elements; (**b**) top view of the fabricated prototype; (**c**) bottom view of the design layout showing the corporate feed network; (**d**) bottom view of the fabricated prototype.

**Figure 3:**
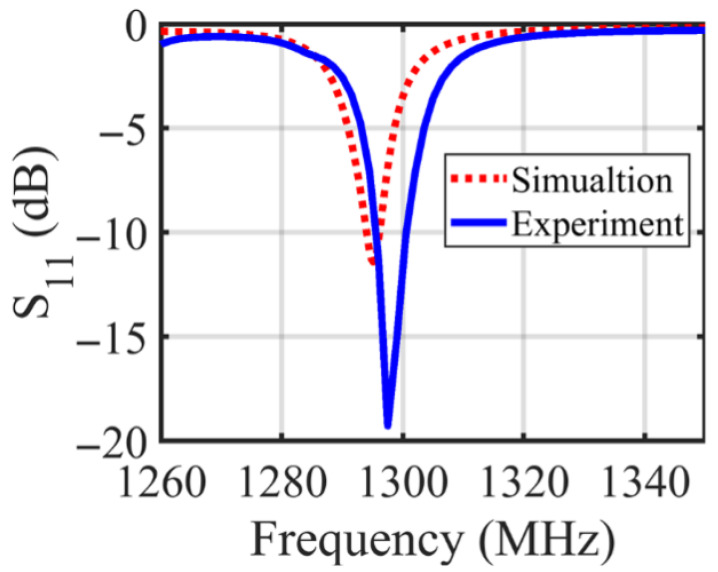
Simulated and measured reflection coefficient ($S_{11}$) of the proposed metasurface array sensor.

The proposed near-field metasurface sensor comprises a periodic 8×8 array of identical unit cells on a dielectric substrate. The sensor array was fabricated according to the proposed design, as shown in Figure 2. Figure 2a and Figure 2b show the top-side view of the designed and fabricated sensor respectively. Figure 2c,d, show the bottom-side view that contains the third layer, which excites the array via a corporate feed network with a single port that uniformly distributes power to all 64 elements. A shared ground plane on the second layer supports both the resonators and the corporate feed network. To match the coaxial 50Ω feed to the corporate network, a single-stub matching circuit is used. Figure 3 shows a comparison between the simulated and measured reflection coefficients ($S_{11}$) of the proposed sensor. The results show good agreement, with a slight frequency shift in the measured data.

## 3. Simulation Validation of the Metasurface Sensor Using Realistic Breast Phantoms

**Figure 4:**
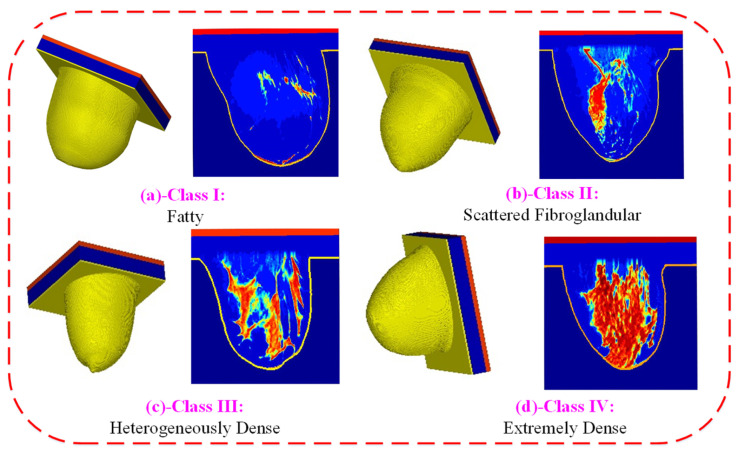
The four numerical breast phantom classes used for sensor evaluation: (**a**) Class I (Fatty), (**b**) Class II (Scattered Fibroglandular), (**c**) Class III (Heterogeneously Dense), and (**d**) Class IV (Extremely Dense). These categories, based on radiographic density, illustrate the variation in breast composition across the patient population. The color visualizations show the permittivity grid for the reconstruction where the adipose (blue) and fibroglandular (red/orange) regions of the heterogeneous interior of the phantoms.

The sensor’s performance was evaluated using breast phantoms that emulate the four breast density categories defined by the American College of Radiology (ACR). These categories, detailed in Figure 4 are: Class I (predominantly fatty), Class II (scattered fibroglandular), Class III (heterogeneously dense), and Class IV (extremely dense). By testing across this spectrum of tissue-mimicking compositions, we comprehensively assessed the sensor’s capability for abnormality detection.

This study utilized anatomically realistic 3D numerical breast phantoms, which were developed in CST Studio Suite [23,24] from breast MRI datasets obtained from the University of Wisconsin’s online repository. The models represent all four American College of Radiology density classes and were created in both normal and abnormal configurations, with the latter incorporating spherical tumors. The dielectric properties were assigned using a piecewise-linear mapping from T1-weighted MRI voxel intensity to the corresponding dielectric characteristics of breast tissues [25,26].

The phantom is a numerical 3D model, where the dielectric properties are derived from T1-weighted MRI images via a piecewise-linear mapping that links voxel intensity to tissue dielectric characteristics [25,26,27]. According to the American College of Radiology (ACR), breast phantoms are classified into four categories based on the radiographic density of fibroglandular tissue: almost entirely fatty, scattered fibroglandular, heterogeneously dense, and very dense [27,28]. For this study, a heterogeneously dense breast phantom with ID: 062204 and ACR Class 3 was employed. The model has a spatial resolution of 0.5×0.5×0.5 mm^3^, with 219×243×273 voxels, as illustrated in Figure 4 After preprocessing the MRI data in MATLAB R2023b, the model includes both the breast volume information and tissue dielectric properties described using the single Cole–Cole model, which is expressed as:

(1) $$\epsilon \left(\omega \right)=\epsilon^{\prime }\left(\omega \right)-j\epsilon^{\prime \prime }\left(\omega \right)=\epsilon_{\infty }+\frac{\Delta \epsilon }{1+{\left(j\omega \tau \right)}^{1-\alpha }}+\frac{\sigma_{s}}{j\omega \epsilon_{o}}$$

where $\epsilon^{\prime }\left(\omega \right)$ is the frequency-dependant relative permittivity, $\epsilon^{\prime \prime }\left(\omega \right)$ is the frequency-dependant dielectric losses, ω is the angular frequency, and $\epsilon_{o}$ is the free-space permittivity. The $\epsilon_{\infty }$, $\sigma_{s}$, τ, and α are the parameters of the single Cole–Cole model obtained from the clinical experimental data [25].

**Figure 5:**
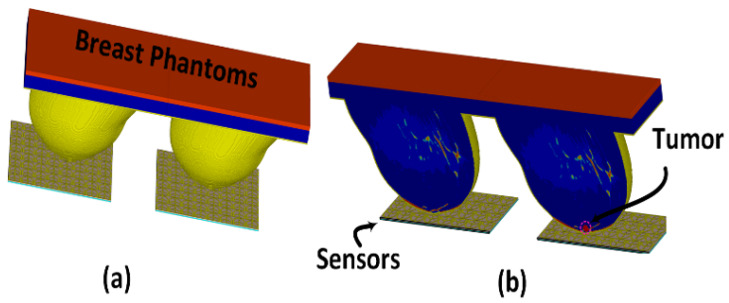
Simulation setup: (**a**) 3D view of the normal (right) and abnormal (left) breast phantoms with the sensor arrays and (**b**) cross-sectional view showing the 5 mm separation between the sensors and phantom surfaces.

The development of an 8×8 metasurface array significantly enhances the sensing area by providing nearly uniform sensitivity across the entire array, which is achieved through strong electromagnetic coupling between the sub-wavelength unit cells. In this context, the sensitivity area is defined as the region over which the sensor can effectively detect perturbations in its near-field distribution, which is manifested as variations in the magnitude and phase of the reflection coefficient S11 across frequency. This strong coupling increases the interaction between the metasurface and breast tissue, enabling the proposed sensor to detect breast abnormalities without any mechanical movement or scanning, as the array effectively covers the entire breast volume within the sensing region (near-field region). It is important to note that the metasurface elements are excited through a corporate feed network, which ensures uniform power distribution to all array elements. Consequently, the proposed sensor is sensitive not only to variations in the electromagnetic properties of the surrounding medium but also to the spatial location at which these variations occur. For example, the observed resonance frequency shift in the presence of a breast tumor, compared to a healthy case, can be attributed to modifications in the current distribution on the sensor when the breast is positioned within the near-field region of the sensor array, as illustrated in Figure 5.

**Figure 6:**
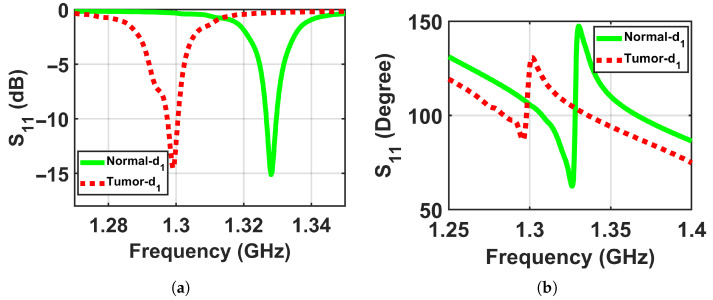
Simulation results showing the sensor responses ($S_{11}$) for normal and abnormal breast phantoms at a stand-off distance of $d_{1}$ = 5 mm. (**a**) Magnitude and (**b**) phase.

**Figure 7:**
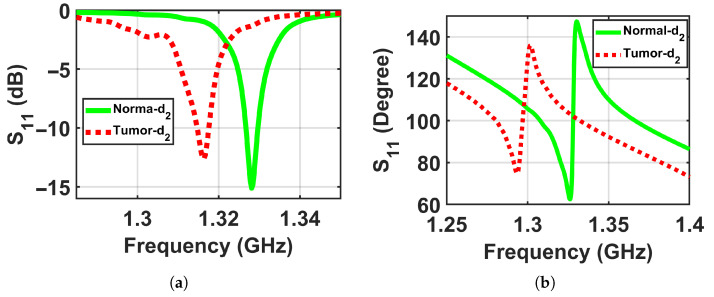
Simulation results showing the sensor responses ($S_{11}$) for normal and abnormal breast phantoms at a stand-off distance of $d_{2}$ = 10 mm. (**a**) Magnitude and (**b**) phase.

**Figure 8:**
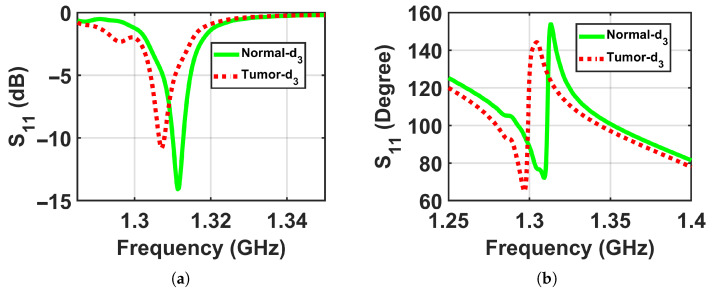
Simulation results showing the sensor responses ($S_{11}$) for normal and abnormal breast phantoms at a stand-off distance of $d_{3}$ = 15 mm. (**a**) Magnitude and (**b**) phase.

To validate the concept of near-field sensing concept of the proposed sensor, where the sensor sensitivity changes based on the distance between the sensor and the breast model, simulation tests were performed on two breast phantoms (normal and abnormal) at three distances off, which were labeled as d1, d2, and d3, where the closest location from the sensor was labeled d1 = 5 mm, the next one further away was labeled d2 = 10 mm, and the farthest was labeled d3 = 15 mm to ensure that the breast model was placed in the sensor’s near-field region and the sensor signals interacted with the entire breast tissues, as shown in Figure 5. The metasurface sensor responses were recorded over a frequency range of both breast models, normal and abnormal, at these three standoff distances. The sensor’s scattering parameters (S11), including magnitudes and phases, were then recorded with a range of frequencies of these three distances off. The data obtained were used to determine whether a tumor was present or not by analyzing the changes in the sensor response (magnitude and phase) with and without the existence of a tumor, as shown in Figure 6, Figure 7 and Figure 8.

**Figure 9:**
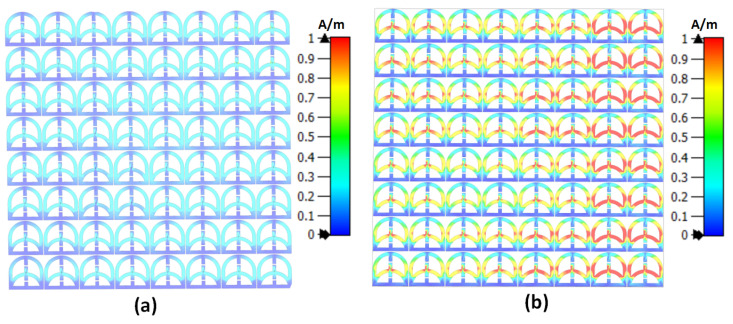
Simualtion result showing surface current distribution on the surface of the metasurface sensor. (**a**) Metasuface sensor with normal breast phantom (without a tumor) and (**b**) metasuface sensor with abnormal breast phantom (with a 10 mm tumor). The blue color corresponds to 0 A/m, and the red color corresponds to 1 A/m.

The novelty of the proposed metasurface sensor stems from its collective electromagnetic behavior, where each sub-wavelength unit cell is strongly coupled to the entire array, such that a localized perturbation in a single cell modifies the global surface current distribution and, consequently, the overall input impedance of the sensor. This principle is clearly illustrated in Figure 9a and Figure 9b, which show the surface current distributions for a normal breast phantom and an abnormal phantom containing a 10 mm tumor, respectively. In the healthy case, the induced currents are uniformly distributed across the metasurface, whereas the presence of a tumor locally alters the dielectric loading, leading to pronounced current redistribution that propagates throughout the entire 8×8 array due to strong inter-element coupling that effects current perturbations that alters the array’s input impedance, leading to corresponding changes in the reflection coefficient S11. Owing to the presence of 64 coupled resonant elements, the metasurface sensor can exhibit a wide range of reflection coefficient variations associated with surface current changes across the entire array. From a metasurface physics perspective, this collective response ensures that even a small dielectric anomaly affects the effective impedance boundary condition of the array, resulting in a measurable shift in the reflection coefficient (S11). Unlike conventional single-resonator sensors, the proposed architecture exploits the impedance sensitivity of a coupled resonant surface, where localized dielectric changes are amplified into global electromagnetic signatures. This mechanism enables high sensitivity without mechanical scanning, as the metasurface intrinsically maps near-field perturbations into detectable reflection coefficient variations across frequency.

## 4. Machine Learning-Based Tumor Detection Using Metasurface Sensor Data

**Figure 10:**
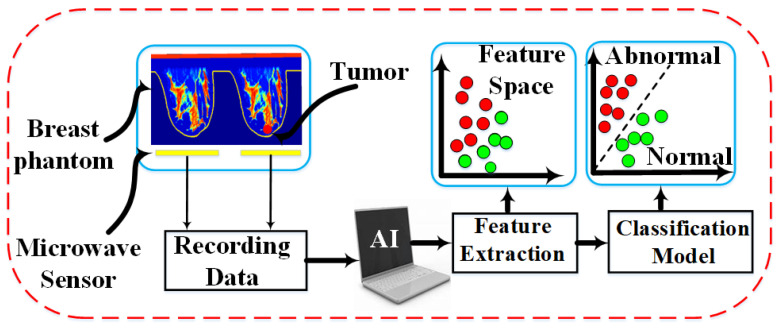
Schematic overview of the detection framework. The system encompasses the numerical phantom setup and sensor data recording (Section 3), followed by the AI processing stages detailed in Section 4, including feature space analysis and the final classification model where red and green dots represent abnormal and normal samples, respectively.

This section details the artificial intelligence framework developed for automated tumor detection using microwave sensor data. As shown in Figure 10, the proposed AI pipeline transforms raw sensor measurements into reliable classification outcomes through a systematic process of data preparation, feature engineering, and machine learning.

### 4.1. Data Distribution and Dataset Composition

The sensitivity of the proposed sensor for breast tumor detection was evaluated through extensive simulations across all four ACR density categories. Sensor responses (both magnitude and phase of ($S_{11}$)) were recorded from 1300 MHz to 1450 MHz for normal and abnormal tissue configurations. The resulting dataset comprises 137 samples organized hierarchically by density class, with each class subdivided into normal and abnormal categories containing varying phantom and tumor sizes, as illustrated in Figure 10. To mitigate overfitting from skewed class distributions, randomization techniques were applied during training to ensure balanced representation across all categories. This approach ensures that all classes are equally represented throughout the training process, promoting a more balanced and generalized model. A total of 137 distinct breast models, encompassing all four classes in both normal and abnormal configurations, were analyzed. In the simulation setup, two sensors were positioned 5 mm from the breast surface opposing the normal (right) and abnormal (left) phantoms, as depicted in Figure 5. The magnitude and phase of the sensor response were recorded for each model to evaluate performance across a wide range of tissue conditions.

### 4.2. Dataset Composition

Figure 6 exemplifies the dataset by presenting the sensor’s magnitude and phase responses for a Class 3 heterogeneously dense phantom, comparing the normal (right) and abnormal (left) configurations. This representative example illustrates the differential response that forms the basis of detection across all four density classes.

Analysis of the initial dataset revealed a compositional bias, consisting primarily of differential measurements between normal and tumorous tissue (Normal–Tumor). This presents a significant limitation, as it fails to represent realistic clinical scenarios involving bilateral screening, where comparisons could be Normal–Normal, or cases of bilateral disease, requiring Tumor–Tumor comparisons. To address this gap and enhance the model’s clinical applicability, a comprehensive data augmentation strategy was implemented. This involved synthetically generating the missing comparative scenarios, which include the following:

- Normal–Normal: Comparison between two normal samples.
- Tumor–Tumor: Comparison between two tumorous samples.

This compination was applied systematically across all four ACR density classes. Consequently, the enhanced dataset now encompassed a more realistic and comprehensive set of classification scenarios: Normal–Normal, Normal–Tumor, Tumor–Normal, and Tumor–Tumor. This approach ensured that the model was exposed to a wider spectrum of potential clinical presentations, significantly improving its robustness and generalizability. The dataset was enhanced to incorporate a more comprehensive set of class labels, representing comparisons across the four American College of Radiology (ACR) breast density classes (C1: fatty, C2: scattered fibroglandular, C3: heterogeneously dense, C4: extremely dense), as detailed in Table 1. Following preprocessing, the entire dataset was randomized and partitioned into training, validation, and testing sets; this data distribution is presented in Table 2.

In this study, we adopted a custom dual-branch neural network tailored for the bilateral comparative analysis of microwave sensor data obtained from left and right breast phantoms, closely aligning with clinical practices that identify tumor-induced asymmetries. The proposed architecture processes frequency, S11 magnitude, and phase information through parallel branches composed of fully connected layers with progressively decreasing sizes (1001, 500, 333, and 263 neurons) and ReLU activation functions. The extracted features are then fused into a 1583-neuron feature vector for robust classification, enabling the capture of subtle dielectric variations that may be overlooked by generic models.

### 4.3. Machine Learning Model Architecture

**Figure 11:**
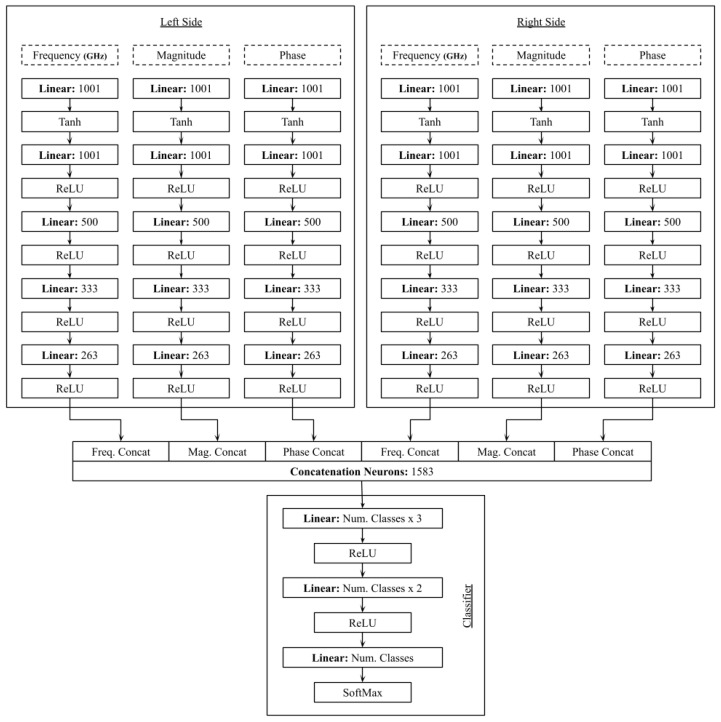
Architecture of the comparator model for analyzing left- and right-side samples.

This study introduces a specialized dual-branch comparator model made to analyze differential measurements between left- and right-side breast phantom samples. The model is designed to perform various tumor classification compositions. This varies from simple normal vs tumor classification across one modality to all four ACR density classes. The complete architecture is illustrated in Figure 11. The model processes left- and right-side samples through two identical, parallel branches. Each branch takes three parallel input streams corresponding to the fundamental sensor parameters: frequency (GHz), magnitude ($S_{11}$), and phase ($S_{11}$). Each parameter is processed independently through a dedicated pathway. The processing begins with a linear layer (1001 neurons) followed by a Tanh activation function, which serves as a feature initialization stage. The data then flow through a sequence of linear layers with progressively reducing dimensionality (1001, 500, 333, and 263 neurons), each followed by a ReLU activation function for non-linear transformation and feature abstraction. A critical step in the architecture is the cross-branch concatenation. Instead of merging all features at once, the model first concatenates the corresponding processed features from both sides, forming three unified representations: a fused frequency vector, a fused magnitude vector, and a fused phase vector. These three fused vectors are then concatenated into a single, comprehensive feature vector of 1583 neurons. This hierarchical fusion strategy explicitly preserves the comparative nature of the data. The final classifier module consists of a stack of linear layers (of size Num. Classes Classes × 3, Num. Classes Classes × 2, and Num. Classes) with ReLU activations, culminating in a SoftMax output layer. This structure allows the model to jointly learn the discriminating features for both the binary (tumor detection) and multi-class (density classification) tasks from the fused comparative representation. The architecture progresses by merging the six independently processed feature streams (three per side) into a consolidated vector of 1583 dimensions. This concatenation layer is pivotal, as it fuses the distinct feature sets from bilateral measurements, establishing a comprehensive input for the subsequent classification stage. After each of the six feature streams (three from each side) are processed separately, they are combined into a single 1583-dimensional vector. This merging step produces a detailed bilateral representation, allowing the model to capture complex relationships among all features for further analysis.

### 4.4. Model Training

The dataset was preprocessed using a custom parser to handle rectangle coordinates, normalization, and data randomization. It was subsequently partitioned into training (70%), validation (15%), and test (15%) subsets to facilitate robust evaluation, as shown in Table 2. The model was trained using the AdamW optimizer, with gradient scaling applied to maintain numerical stability under mixed-precision arithmetic. The training and validation pipelines utilized separate data instances, and shuffling was enabled for the training set to ensure robust batch sampling, while the validation and test sets were processed in their original order. To optimize performance on modern hardware, the model was trained with mixed precision using a gradient scaler, which maintained numerical stability throughout the process, as shown in Table 3.

## 5. Results and Discussion

**Figure 12:**
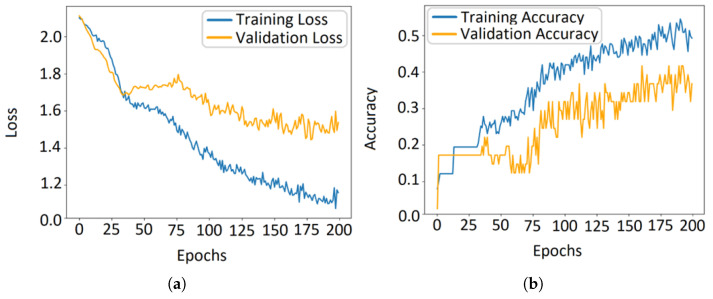
Training performance for normal and tumor classifications. (**a**) Training loss and (**b**) accuracy across all four classes (C1–C4).

The influence of class combination and dataset composition on model convergence is shown in Figure 12. The plots of training loss and accuracy reveal that the discriminability between Class 1, Class 2, Class 3, and Class 4 is not uniform; certain class pairs were learned more rapidly and accurately than others. Furthermore, the performance varied significantly between normal and abnormal phantom types and showed a direct correlation with the available sample size for each condition.

**Figure 13:**
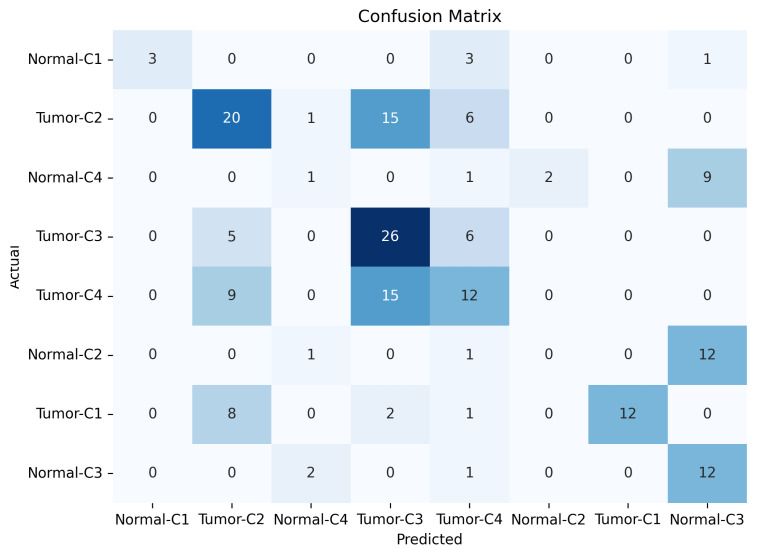
Results of confusion training matrice for normal and tumor classification across four tissue classes (C1–C4).

**Figure 14:**
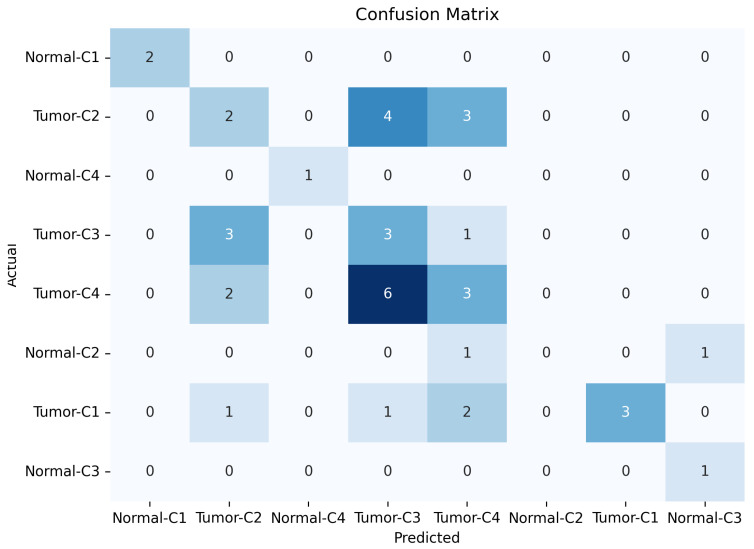
Results of confusion validation matrice for normal and tumor classification across four tissue classes (C1–C4).

**Figure 15:**
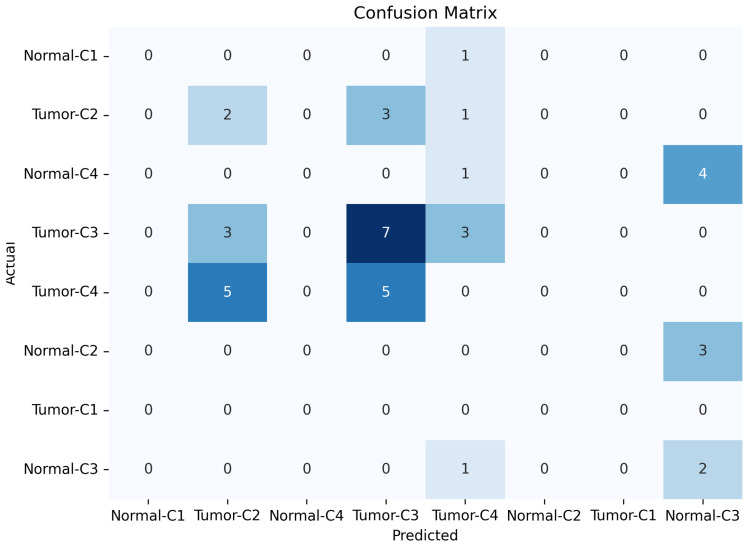
Results of confusion testing matrice for normal and tumor classification across four tissue classes (C1–C4).

Figure 13, Figure 14 and Figure 15 show the training, validation, and testing confusion matrix for the same four-class combinations (Class 1, Class 2, Class 3, and Class 4) across both normal and abnormal breast phantoms. The matrices quantify classification performance and reveal specific patterns of inter-class confusion. In addition, each matrix provides a detailed view of the model’s classification performance, showing how accurately it distinguishes between individual classes. Each matrix also reveals specific misclassifications, highlighting the model’s strengths and weaknesses across different class combinations and phantom conditions. By examining these results, we can identify key opportunities to improve the model’s accuracy and robustness, especially for classes that are more challenging to distinguish.

Table 4 presents the performance metrics for the model classifying normal and tumor categories (Class 1, Class 2, Class 3, and Class 4) across three datasets: training, validation, and testing. The key performance metrics include loss, accuracy, average precision, average recall, and average F1-score. The model achieves its best performance on the training dataset, with an accuracy of 50.27%, an average precision of 0.517, average recall of 0.503, and an average F1-score of 0.490. However, there is evidence of overfitting, as seen in the significant drop in performance on the validation dataset, where the accuracy decreases to 37.5%, and both the precision and recall show a notable decline. On the testing dataset, the model’s performance further declines, with an accuracy of 26.83%, average precision of 0.253, recall of 0.389, and an F1-score of 0.301. These results highlight the model’s limitations and indicate that substantial improvements are needed to enhance its generalization capability and overall performance.

The model’s result across different class combinations show clear strengths, along with room for growth. It does very well with single classes, such as C1 and C2, where it reaches 99% accuracy on training, validation, and testing data, with perfect precision, recall, and F1-scores. C4 also performs strongly, with accuracy from 98% to 99% and solid metric scores, which points to the model’s reliability for simpler tasks.

To mitigate class imbalance while preserving physical realism, we adopted a conservative pairwise data organization strategy using existing, physically simulated breast phantoms within the same ACR density class. No S11 signals were altered or synthesized. This approach allowed more uniform exposure to bilateral comparison scenarios (Normal–Normal, Normal–Tumor, Tumor–Normal, Tumor–Tumor), which supports the intended operation of the asymmetry-based dual-branch architecture. The resulting paired samples are not statistically independent and do not represent the full range of true bilateral anatomical variability. Their role is limited to facilitating stable comparative learning during training and should not be interpreted as a replacement for additional simulations or clinical measurements.

For pairs of classes, like C1–C2 and C2–C3, the model holds up reasonably well, with accuracies of 71% and 65%. This suggests it can manage moderate challenges. As tasks get more complex, such as with C3 alone or all four classes together (C1–C2–C3–C4), the results vary and offer chances for betterment. The strong base from single-class work, and steady results across data types, gives a good starting point. With some adjustments, the model could handle tougher multi-class problems more effectively.

Looking at the model’s performance across class combinations reveals some key trends in the training, validation, and testing metrics. It excels with individual classes C1 and C2, hitting 99% accuracy across all data splits, with perfect precision, recall, and F1-scores. C4 follows closely, with 98% to 99% accuracy and high scores. But C3 performs much weaker, around 25% accuracy across splits.

For two-class pairs, the results differ. C2–C3 gives balanced outcomes, around 65% accuracy. C1–C2 reaches 71% in training but dips in validation. C2–C4 shows the lowest, at 48% in training and less in testing. With three or four classes, the performance generally drops. C1–C2–C3 holds 62% in training but falls in validation and testing. The full four-class set (C1–C2–C3–C4) gets 50% in training but only 26.83% in testing. Loss values rise with more classes, from a low of 0.00089 for singles to 1.5689 for all four. This pattern indicates that adding classes makes separation harder. Overall, while the model shines in simple classifications, it weakens with more classes, highlighting paths for refinement in multi-class settings.

The model demonstrates exceptional performance when trained on individual tissue classes C1 and C2, achieving 99% accuracy across training, validation, and testing splits, with perfect precision, recall, and F1-scores. Class C4 also exhibits strong and consistent performance, with accuracies ranging from 98% to 99% and similarly high evaluation metrics. In contrast, class C3 shows substantially weaker performance, with the accuracy remaining around 25% across all data splits, indicating increased intra-class variability and reduced separability of tumor-induced features for heterogeneously dense breast tissue. When combining two tissue classes, the classification performance varies noticeably depending on the class pairing. The C2–C3 combination yields the most balanced results, with accuracies of approximately 65%, suggesting partial feature overlap that remains moderately distinguishable. The C1–C2 pairing achieves 71% training accuracy but exhibits some degradation in validation performance, indicating limited generalization. The C2–C4 combination performs the poorest among the paired scenarios, with training accuracy decreasing to 48% and further degradation observed in the testing phase. As the classification task becomes more complex with the inclusion of three or more tissue classes, a general decline in performance is observed. The C1–C2–C3 combination maintains moderate training accuracy (approximately 62%) but suffers from significant reductions in validation and testing performance. The full four-class scenario (C1–C2–C3–C4) proves to be the most challenging, achieving only 50% training accuracy and exhibiting substantial inconsistency across validation and testing sets, with test accuracy dropping to 26.83%. This performance trend is further reflected in the loss values, which increase progressively with task complexity. The loss remains very low for single-class experiments (as low as 0.00089) but rises steadily as additional classes are introduced, reaching 1.5689 for the four-class configuration. This behavior highlights the increasing difficulty of learning discriminative features across heterogeneous breast tissue classes.

The multi-class breast density classification (C1–C4) exhibits a marked performance reduction, with accuracy approaching near-random levels, primarily due to strong dielectric overlap between adjacent density classes that results in highly similar S11 responses. This limits reliable separability using a compact model. Accordingly, the principal contribution of this work is binary tumor detection, where consistently high performance is achieved across individual density classes. The multi-class results are included to transparently highlight current limitations rather than to suggest clinical readiness. A hierarchical strategy—tumor detection followed by density classification using larger datasets and higher-capacity models—is a more suitable formulation and is identified as future work.

Overall, these results indicate that the proposed model is highly effective for tumor detection within individual breast tissue classes, where dielectric variability is limited. However, its performance degrades as inter-class heterogeneity increases, underscoring the need for further optimization to improve robustness in multi-class classification scenarios.

## 6. Experiment Validation

**Figure 16:**
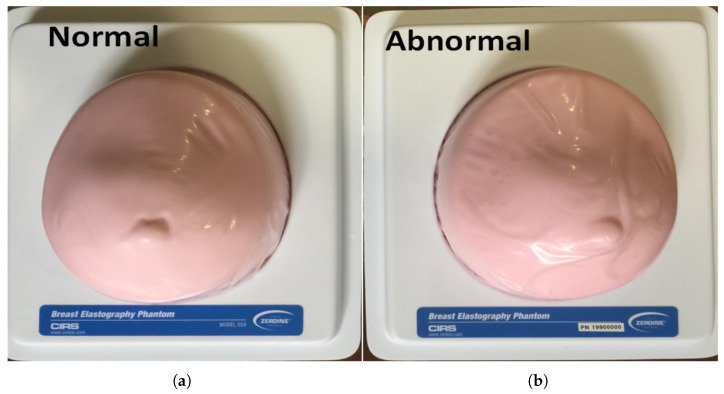
Fabricated breast phantoms: (**a**) normal breast phantom and (**b**) abnormal breast phantom.

To validate the sensitivity of the proposed sensor for detecting breast tumors, the fabricated sensor was tested with two normal and abnormal fabricated breast phantoms, as shown in Figure 16. Standard models of the breast elastography phantoms were used in this study, which were fabricated and designed by the CIRS company (Melbourne, FL, USA) [29]. These phantoms are approximately 15 cm long × 12 cm wide × 7 cm high, with a volume of 600 cc. The abnormal phantom has a 10 mm tumor that is approximately three times harder than the background material and is positioned in the upper half top quarter of the phantom. The tumor has a higher concentration of water than the background gel, which should result in a different conductivity and a dielectric constant higher than normal tissues.

The custom breast phantoms were requested from the manufacturer for proof-of-concept testing of microwave detection. The phantom was designed to replicate heterogeneous breast tissue and include abnormal lesions for elastography and detection validation. In this phantom, the lesions contain a higher water concentration than the surrounding background gel, resulting in increased conductivity and relative permittivity in the microwave frequency range. This difference in water content serves as the primary source of contrast for microwave-based detection. The phantom comprises multiple tissue-equivalent layers, including skin, subcutaneous fat, bulk fat, and glandular/fibroglandular tissues, along with cystic and dense lesions embedded within the breast background. The skin layer is simulated using a patent-pending Z-Skin™ membrane, which protects the phantom from desiccation during repeated use.

The dielectric properties of the phantom materials are designed to match typical breast tissue characteristics in the microwave range (0.5–10 GHz). For example, skin and glandular layers exhibit higher relative permittivity (35–50) and conductivity (0.8–1.5 S/m), whereas adipose/fat layers have lower permittivity (5–15) and conductivity (0.01–0.1 S/m). The contrast in water content between lesions and background provides the electromagnetic distinction necessary for validating microwave detection techniques. By combining anatomical realism with appropriate dielectric properties, this phantom provides a robust platform for experimental proof-of-concept studies.

**Figure 17:**
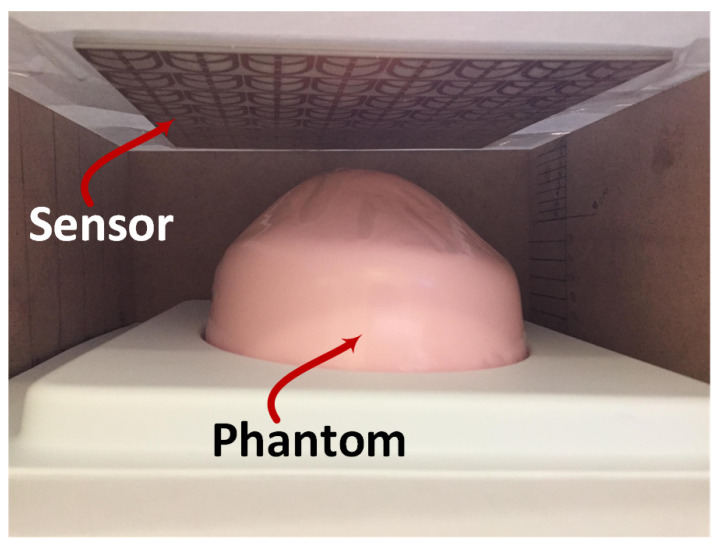
Experiment setup demonstrating the sensor with a breast phantom inside a wooden box.

The experiment setup consisted of the fabricated sensor, a wooden box, a VNA, and breast phantoms, as shown in Figure 17. The experiment procedure was as follows: The sensor was placed at the top of the wooden box, and the breast phantom was placed inside the box to facilitate easy movement or change between the normal and abnormal phantom, as well as to easily change the stand-off distances between the sensor and phantom.

Experimental validation in this study is intended as a preliminary proof of feasibility. Measurements were performed on two fabricated breast phantoms (normal and tumor-bearing) with a single tumor size (10 mm) and repeated at three sensor–phantom stand-off distances (5, 10, and 15 mm). The setup for testing different stand-off distances involved placing the breast phantom on foam support. For each distance, the breast phantom was positioned on a foam with a specific thickness matching the distance being tested, as illustrated in Figure 17.

**Figure 18:**
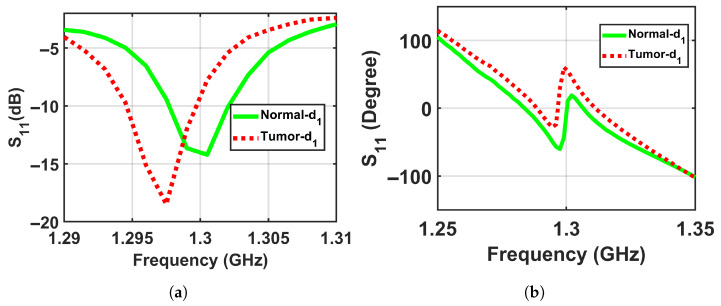
Measurement results showing the sensor response with the 3D breast realistic phantom with and without a 10 mm tumor at a stand-off distance of $d_{1}$ = 5 mm. (**a**) $S_{11}$ magnitude (dB) and (**b**) $S_{11}$ phase (degree).

**Figure 19:**
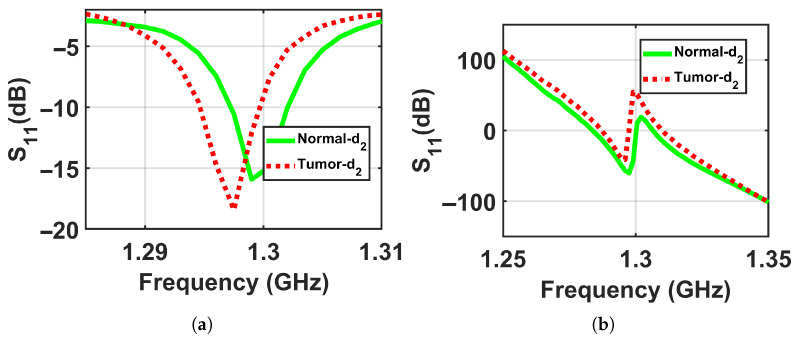
Measurement results showing the sensor response with the 3D breast realistic phantom with and without a 10 mm tumor at a stand-off distance of $d_{2}$ = 10 mm. (**a**) $S_{11}$ magnitude (dB) and (**b**) $S_{11}$ phase (degree).

**Figure 20:**
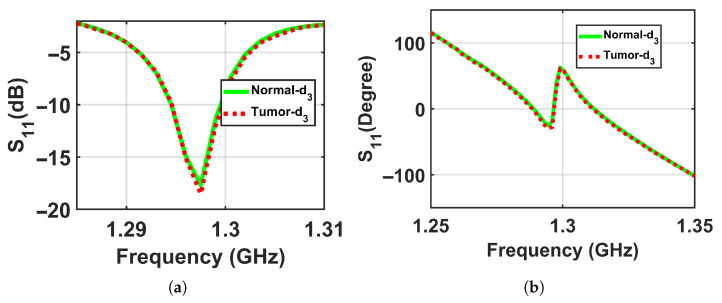
Measurement results showing the sensor response with the 3D breast realistic phantom with and without a 10 mm tumor at a stand-off distance of $d_{3}$ = 15 mm. (**a**) $S_{11}$ magnitude (dB) and (**b**) $S_{11}$ phase (degree).

The results show detectable differences between normal and abnormal cases at shorter distances, with the contrast decreasing at the largest distance, which confirms the physical principle of the proposed sensor and its enhanced sensitivity in the near-field region, as shown in Figure 18, Figure 19 and Figure 20 respectively.

## 7. Conclusions

In this study, a metasurface sensor consisting of 64-unit cells was designed for breast tumor detection. The sensor was tested on breast phantoms with varying tissue types, including fatty, scattered fibroglandular, heterogeneously dense, and extremely dense tissues. Both magnitude and phase responses were recorded from the sensor for normal and abnormal tissue samples. A custom neural network model was developed to compare left and right breast samples, focusing on detecting potential tumors. The model utilized cross-entropy loss for classification and was trained with a CUDA-enabled GPU using the AdamW optimizer for improved performance. The dataset was split into training, validation, and test sets (70-15-15)%, with data normalization, randomization, and rectangle coordinate handling managed by a custom parser. The results demonstrated that the model achieved high accuracy for individual tissue types, reaching up to 99% accuracy in certain cases. However, as more tissue categories were introduced, the performance began to decline. The four-class combination, in particular, resulted in only 50% training accuracy and a significant decrease in testing accuracy, highlighting challenges when handling multi-class scenarios. The model incorporated gradient scaling to prevent numerical instability, especially during mixed precision calculations. These findings reveal that while the sensor and neural network model excel in single-class classification tasks, they face difficulties when tasked with multi-class detection. This suggests that while the sensor is highly effective for identifying individual tissue types, further optimization is required to enhance performance in more complex multi-class scenarios. The insights gained from this study offer valuable direction for the development of electromagnetic scan-based systems for breast tumor detection, opening avenues for future advancements in improving multi-class classification and overall system robustness.

## Figures and Tables

**Table 1:** Class-label distribution after data enhancement.

Sr #	Class-Label Cases for Comparator Model	Number of Samples
1	Normal-C1	10
2	Tumor-C1	30
3	Normal-C2	19
4	Tumor-C2	57
5	Normal-C3	19
6	Tumor-C3	57
7	Normal-C4	19
8	Tumor-C4	57
Total		268

**Table 2:** Data samples distribution for model development and evaluation.

Data Type	Division Percentage (%)	Number of Samples
Training Data	70	187
Validation Data	15	40
Testing Data	15	41
Total	100	268

**Table 3:** Model training hyperparameters.

Parameter	Value	Description
Batch Size	75	Number of samples per training iteration
Epochs	200	Total training cycles
Learning Rate	0.0001	Initial learning rate for optimization
Weight Decay	1 × 10^−4^	L2 regularization factor
Sequence Size	1001	Input sequence length
Device	NVIDIA	Primary GPU device
Data Split	70/15/15	Train/valid/test Ratio
Workers	1	Number of data loading workers

**Table 4:** Model performance evaluation on multiple combinations.

Combination	Data Type	Loss	Accuracy (%)	Avg. Precision	Avg. Recall	Avg. F1-Score
C1	Training	0.00089	99	1.0	1.0	1.0
	Validation	0.00073	99	1.0	1.0	1.0
	Testing	0.0005	99	1.0	1.0	1.0
C2	Training	0.06809	99	1.0	1.0	1.0
	Validation	0.13709	99	1.0	1.0	1.0
	Testing	0.0941	99	1.0	1.0	1.0
C3	Training	0.79829	24.52	0.0	0.0	0.0
	Validation	0.78849	27.27	0.0	0.0	0.0
	Testing	0.7965	25	0.25	1.0	0.4
C4	Training	0.22601	98.113	0.976	1.0	0.988
	Validation	0.27008	90.909	0.89	1.0	0.94
	Testing	0.2032	99	1.0	1.0	1.0
C1, C2	Training	0.71944	71.604	0.808	0.741	0.719
	Validation	0.85469	58.823	0.67	0.588	0.543
	Testing	0.7528	72	0.796	0.722	0.734
C1, C3	Training	0.83756	62.962	0.444	0.500	0.461
	Validation	1.28624	41.176	0.170	0.412	0.240
	Testing	0.8463	55.56	0.443	0.556	0.467
C1, C4	Training	0.78451	60.493	0.789	0.654	0.638
	Validation	0.98267	64.705	0.824	0.647	0.670
	Testing	0.9827	64.71	0.527	0.656	0.536
C2, C3	Training	0.62034	66.037	0.687	0.679	0.673
	Validation	0.68133	63.636	0.723	0.636	0.625
	Testing	0.8391	66.67	0.710	0.667	0.684
C2, C4	Training	0.87645	48.113	0.524	0.472	0.419
	Validation	1.05027	36.363	0.451	0.364	0.349
	Testing	0.9996	29.17	0.2315	0.2917	0.2581
C3, C4	Training	0.73128	63.207	0.629	0.642	0.618
	Validation	0.99167	50.0	0.422	0.449	0.401
	Testing	0.9451	50	0.542	0.500	0.488
C1, C2, C3	Training	0.92764	62.686	0.634	0.657	0.628
	Validation	1.16177	39.285	0.396	0.321	0.303
	Testing	1.0806	43.33	0.433	0.500	0.464
C1, C2, C4	Training	1.00694	55.9701	0.580	0.602	0.573
	Validation	1.26116	39.285	0.121	0.263	0.165
	Testing	1.5184	40	0.599	0.429	0.448
C1, C3, C4	Training	1.00694	55.9701	0.644	0.627	0.624
	Validation	1.26116	39.285	0.520	0.538	0.515
	Testing	1.5184	40	0.235	0.267	0.240
C2, C3, C4	Training	1.39604	37.10691	0.477	0.409	0.360
	Validation	1.41714	35.29411	0.588	0.353	0.308
	Testing	1.3815	25.71	0.351	0.257	0.243
C1, C2, C3, C4	Training	1.15911	50.2673	0.517	0.503	0.490
	Validation	1.5376	37.5	0.480	0.429	0.438
	Testing	1.5689	26.83	0.253	0.389	0.301

## Data Availability

The raw data supporting the conclusions of this article will be made available by the authors on request.
